# Activation of a PAK-MEK signalling pathway in malaria parasite-infected erythrocytes

**DOI:** 10.1111/j.1462-5822.2011.01582.x

**Published:** 2011-06

**Authors:** Audrey Sicard, Jean-Philippe Semblat, Caroline Doerig, Romain Hamelin, Marc Moniatte, Dominique Dorin-Semblat, Julie A Spicer, Anubhav Srivastava, Silke Retzlaff, Volker Heussler, Andrew P Waters, Christian Doerig

**Affiliations:** 1 INSERM U609/Inserm-EPFL Joint Laboratory, Global Health InstituteCH-1015 Lausanne, Switzerland; 2 Proteomics Core Facility, Ecole Polytechnique Fédérale de LausanneCH-1015 Lausanne, Switzerland; 3 Auckland Cancer Society Research Centre, Faculty of Medical and Health Sciences, The University of AucklandPrivate Bag 92019, Auckland 1142, New Zealand; 4 Division of Infection and Immunity, Faculty of Biomedical Life Sciences, & Wellcome Trust Centre for Molecular Parasitology, Glasgow Biomedical Research Centre, University of Glasgow120 University Place, Glasgow G12 8TA, Scotland, UK; 5 Bernhard Nocht Institute for Tropical MedicineBernhard-Nocht-Str. 74, 20359 Hamburg, Germany; 6 Institute of Cell Biology, University of BernBaltzerstasse 4, 3012 Bern, Switzerland; 7 Wellcome Trust Centre for Molecular Parasitology, University of GlasgowGlasgow G12 8TA, Scotland, UK

## Abstract

Merozoites of malaria parasites invade red blood cells (RBCs), where they multiply by schizogony, undergoing development through ring, trophozoite and schizont stages that are responsible for malaria pathogenesis. Here, we report that a protein kinase-mediated signalling pathway involving host RBC PAK1 and MEK1, which do not have orthologues in the *Plasmodium* kinome, is selectively stimulated in *Plasmodium falciparum*-infected (versus uninfected) RBCs, as determined by the use of phospho-specific antibodies directed against the activated forms of these enzymes. Pharmacological interference with host MEK and PAK function using highly specific allosteric inhibitors in their known cellular IC_50_ ranges results in parasite death. Furthermore, MEK inhibitors have parasiticidal effects *in vitro* on hepatocyte and erythrocyte stages of the rodent malaria parasite *Plasmodium berghei*, indicating conservation of this subversive strategy in malaria parasites. These findings have profound implications for the development of novel strategies for antimalarial chemotherapy.

## Introduction

Many intracellular pathogens, including unicellular eukaryotic parasites, tailor their immediate environment to their specific needs by affecting the properties of their host cell (Haldar and Mohandas, 2007). Sporozoites of malaria parasites ensure survival of their host hepatocyte by preventing apoptosis and inflammation, through interference with host cell NF-κB and HGF pathways (Heussler *et al*., 2006; Singh *et al*., 2007). Additional host hepatocyte signalling protein kinases have been implicated in *Plasmodium* liver-stage development (Prudencio *et al*., 2008). *Plasmodium* relies on host heterotrimeric G-proteins for the establishment of red blood cell (RBC) infection (Harrison *et al*., 2003), suggesting the existence of an interface between the parasite and host cell signalling elements in blood stages as well. Even though RBCs have no need for signalling pathways regulating gene expression or cell proliferation, signalling elements including MAPK (mitogen-activated protein kinase) modules are nevertheless maintained (Ringrose *et al*., 2008), with proposed functions in the regulation of ion transport (Sartori *et al*., 1999) or membrane mechanical properties (Manno *et al*., 2005). At the core of the MAPK pathways lies a three-component module comprising the MAPK itself (also called ERK, for extracellularly regulated kinase), its activator the MAPKK (MEK, for MAPK/ERK kinase) and a third, yet more upstream kinase, the MAPKKK or MEKK (Raman *et al*., 2007). MEKK-independent MEK activation, notably through the p21-activated protein kinase PAK, has also been characterized (Slack-Davis *et al*., 2003; Park *et al*., 2007). The *Plasmodium* kinome encodes two divergent MAPKs (Dorin-Semblat *et al*., 2007), but clearorthologues of mammalian MEK1/2 and PAK have not been identified in the parasite (Ward *et al*., 2004; Dorin *et al*., 2005). Here we show that a host erythrocyte signalling pathway involving MEK1 and PAK1 is stimulated by *Plasmodium falciparum* infection, and that pharmacological interference with this pathway using highly specific allosteric inhibitors of human PAK and MEK enzymes causes a block in parasite proliferation in both liver and blood stages of mammalian host infection.

## Results and discussion

### Pharmacological evidence that host MEK activity is required for parasite survival

**Fig. 1:**
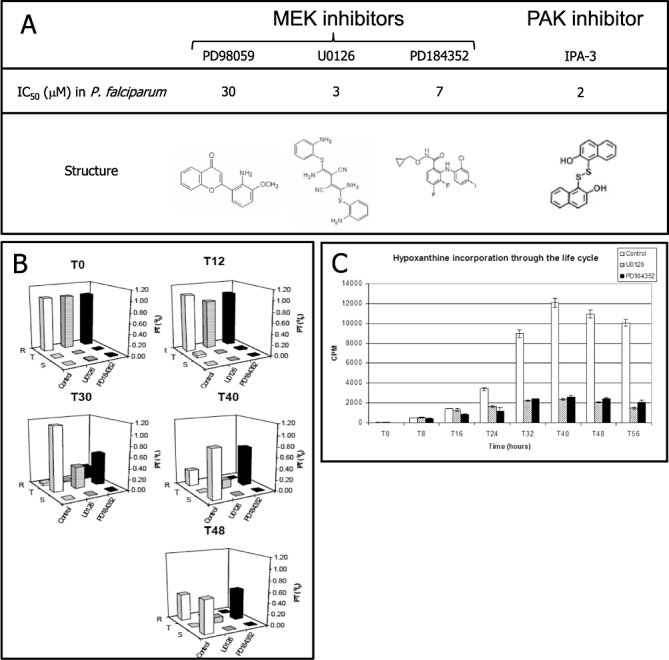
Effect of MEK inhibitors on the *P. falciparum* erythrocytic asexual cycle. A. Structure of the MEK inhibitors used in this study. The IC_50_ values on *P. falciparum* growth are indicated above the structures. B. MEK inhibitors block trophozoite development. Synchronized cultures (2% parasitaemia) were treated at the ring stage with MEK inhibitors (U0126 and PD184352, 20 µM), and aliquots were smeared at 0, 12, 30, 40 and 48 h post treatment. Cell numbers were obtained from microscopic examination of 10 fields for each time point. The experiment was performed three times in triplicate, with similar results. C. MEK inhibitors impair parasite DNA synthesis. Hypoxanthine incorporation along *P. falciparum* life cycle was measured in the presence of MEK inhibitors (U0126 and PD184352, 20 µM). MEK inhibitors (or DMSO as a negative control) and [^3^H]-hypoxanthine were added to tightly synchronized cultures (3% parasitaemia, ring stage) at T0. The cells were then harvested at 8 h intervals, and precipitable tritium was quantified by scintillation. Error bars show the SEM. The experiment was performed twice in triplicate, with similar results.

In the course of our investigations on *Plasmodium* MAPK pathways, we found that the highly selective MEK1/2 inhibitor U0126 inhibited *P. falciparum* proliferation, with an IC_50_ value of 3 µM (Fig. 1A; see Fig. S1A for IC_50_ determination data) comparable to the 2 µM IC_50_ value of the compound in a mammalian T cell proliferation assay (DeSilva *et al*., 1998). This is surprising, in view of the absence of typical MEK homologues in the *Plasmodium* kinome (Ward *et al.*, 2004). Furthermore, the structurally distinct allosteric MEK inhibitors PD98059 and PD184352 (also known as CI-1040) also had parasiticidal activity, with IC_50_ values of 30 and 7 µM respectively (Fig. S1A). The former value (for PD98059) is within the range of known IC_50_ in various cell systems; the latter (for PD184352) is similar to, but somewhat higher than, those observed in mammalian cell assays: for example, PD184352 causes cell cycle arrest of fibroblasts with an IC_50_ of ∼1 µM (Squires *et al*., 2002). Treatment of synchronized parasite populations with U0126 and PD184352 showed that both inhibitors block trophozoite development (Fig. 1B), and that DNA synthesis is impaired in parasites from treated cultures (Fig. 1C). In contrast, when the inhibitors were added to synchronized cultures of mature schizonts, we observed that egress, invasion and establishment of the ring stage were unaffected, as indicated by similar parasitaemia in the newly infected erythrocytes in all samples (Fig. S2).

**Fig. 2:**
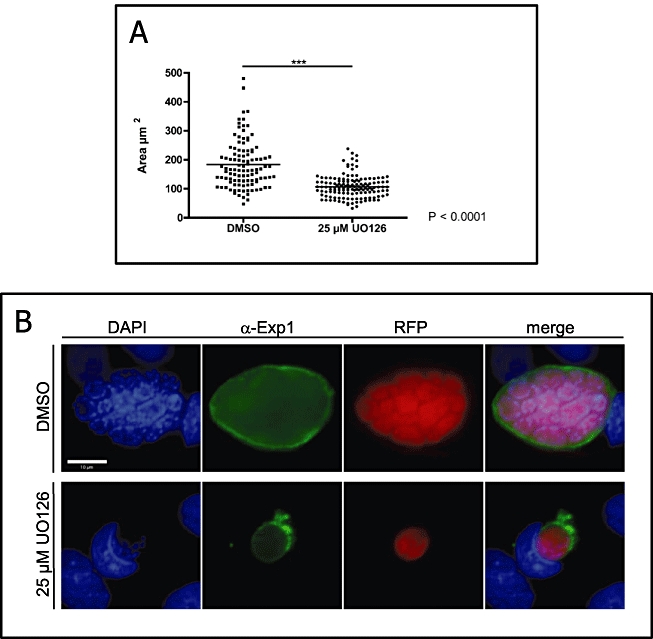
Effect of MEK inhibitors on *P. berghei* erythrocytic and liver stages. A. HepG2 cells were infected with *P. berghei* expressing RFP under the control of the promoter region of the constitutively expressed *P. berghei* eukaryotic elongation factor 1 alpha (eef1aa). Twenty-four hours post infection, infected cell cultures were treated with 25 µM UO126 for another 24 h or were left untreated as a control. RFP fluorescence of parasites was monitored by live imaging; the size of the parasite was quantified and expressed in µm^2^. Statistical evaluation using the Student's *t*-test revealed a highly significant difference in parasite size (*P*: 0.0001). B. Immediately after live imaging, cells were fixed and stained with an antiserum directed against the PVM protein Exp1 (green). DNA was visualized with DAPI (blue). Parasites were still clearly visible by the remaining RFP fluorescence (red). Merged pictures are presented on the right. Representative images of treated (U0126) and untreated (DMSO) cells are presented. This is one of several similar pictures obtained with treated cells.

MEK inhibitors also have parasiticidal activity against the rodent malaria parasite *Plasmodium berghei*, which belongs to a distinct subgroup of the *Plasmodium* genus: the sensitivity of *P. berghei* to PD184352 has a similar level (IC_50_ = 8.3 µM, Fig. S3) and stage specificity (block of trophozoite maturation, not shown) as that of *P. falciparum* (IC_50_∼7 µM). Using a transgenic parasite line expressing RFP (Graewe *et al*., 2009), we also demonstrated an effect of 25 µM U0126 on the development of *P. berghei* hepatocytic schizonts in HepG2 cells: intracellular parasites within treated cells were significantly smaller than those in untreated cultures (Fig. 2A and B), demonstrating that U0126 treatment clearly impairs parasite growth and development of liver-stage parasites. Interestingly, in the host kinome-wide siRNA knock-down experiment reported by Prudencio *et al*. (2008) (see Table S1 of this article), both a PAK isoform (see below) and a MEK-interacting protein were identified as having a detectable effect on liver infection.

**Fig. 3:**
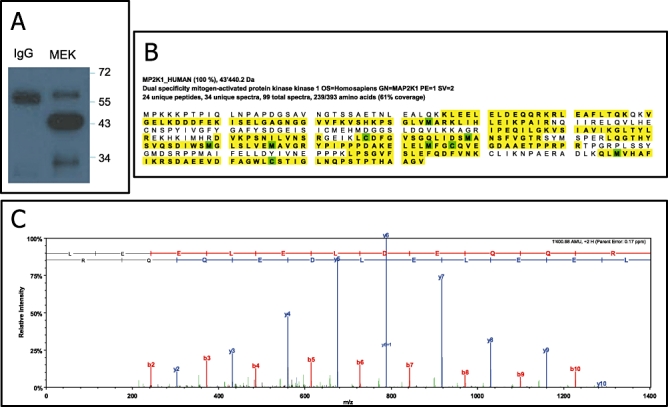
Identification of MEK1 in the erythrocyte. A. Western blot of total-cell extracts from uRBCs after immunoprecipitation using either mouse anti-MEK1 agarose-conjugated or mouse IgG agarose-conjugated as a control probed with an anti-MEK1 antibody. B. Sequence Coverage of the identified MEK1 protein in erythrocyte. C. Representative CID fragmentation pattern of a unique peptide identified in MEK1.

Most protein kinase inhibitors target the ATP-binding pocket and therefore tend to be poorly selective (Davis, 2000; Bain *et al*., 2007). In contrast, the allosteric MEK inhibitors used in these experiments bind to a MEK-specific pocket that is distinct from the ATP-binding pocket, and freeze the enzyme in the inactive conformation (Ohren *et al*., 2004). This class of inhibitors thus displays exquisite selectivity against MEK1 and MEK2, with lower activity against MEK5 as well (Bain *et al*., 2007). Since the parasite's kinome does not include orthologues of these enzymes, this strongly suggests that in our experiments their target is a human MEK rather than a parasite-encoded protein. Mass spectrometry analysis of an immunoprecipitate obtained with anti-human MEK1 antibodies from an uninfected human RBC extract (Fig. 3A) identified 24 unique MEK1 peptides with a coverage of 61% (Fig. 3B and C; see Fig. S4 for a list of peptides), validating a previous mention (Roux-Dalvai *et al*., 2008) of the presence of this enzyme in uninfected erythrocytes.

### Phosphorylation of MEK1 in infected erythrocytes

**Fig. 4:**
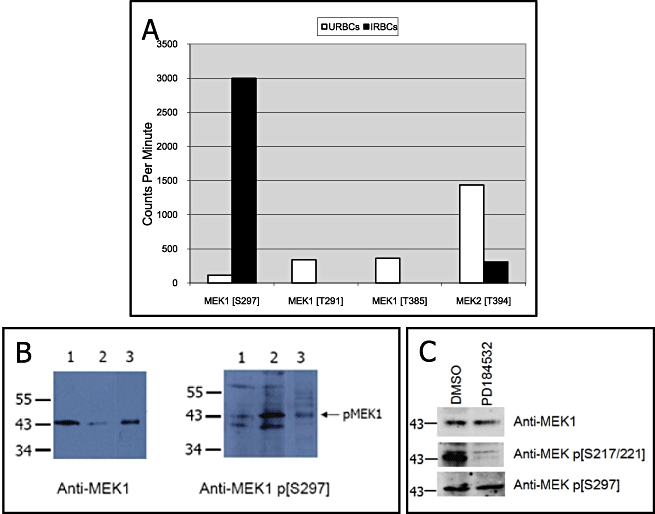
Phosphorylation status of host cell MEK1 in *P. falciparum*-infected erythrocytes. A. Quantification of Western blot data obtained from Kinexus experiment (see text for details and Fig. S5 for the original Western blot). The autoradiogram was scanned to obtain a counts per minute (c.p.m.) value (scan time of 60 s). Data in Figs 4A and S5 were generated at Kinexus (Vancouver, Canada) using cell extracts provided by the authors. B. Western blot of total-cell extracts from uRBCs (lane 1) and iRBCs (lane 2). Lane 3 is a positive control extract (3T3 cells treated with PDGF) provided by the supplier of the antibodies (Biosource). The membrane was probed first with an anti-MEK1 antibody recognizing both phosphorylated and non-phosphorylated forms of the protein (left panel). The same membrane was then probed with the anti-phospho-MEK1 p[S297] (right panel). C. Effect of the PD184532 MEK inhibitor on MEK phosphorylation. Synchronous *P. falciparum* cultures were treated at ring stage with 20 µM PD184532 (or with the DMSO vehicle only) for 24 h prior to Western blot analysis using the antibodies indicated to the right.

To detect a possible effect of infection on host MEK activation, we used the KPSS7.0 panel of antibodies from Kinexus Corporation (Vancouver, Canada), which contains a number of phospho-specific antibodies recognizing a panel of signalling molecules and covering several phosphorylation sites on MEK1 and MEK2. Extracts from uRBCs and iRBCs, normalized for cell number, were subjected to Western blot analysis using the KSSP7.0 panel. Strikingly, a very strong signal was obtained with the antibody against p[Ser-297] of human MEK1 only in the iRBC sample (Fig. 4A; see Fig. S5 for the original Western blot from Kinexus). We ensured that MEK1 protein levels were similar in both samples, by performing a Western blot analysis using first an antibody against MEK1, and then, on the same membrane, the p[Ser-297] antibody. Both iRBC and uRBC extracts contained MEK1 protein; indeed, MEK1 was more abundant in the uRBC extract. However, the signal yielded by the p[Ser-297] antibody was stronger in iRBCs than in uRBCs, despite a lower amount of protein (Fig. 4B). This confirms that MEK1 Ser-297 phosphorylation is strongly stimulated in infected erythrocytes. In contrast to the much stronger MEK1 p[Ser-297] signal in infected (versus uninfected) cells, there was a decrease in the phosphorylation in iRBCs (versus uRBCs) of two other residues in MEK1 (Thr-291 and Thr-385), as well as MEK2 Thr-394 (Fig. 4A). Phosphorylation of these three residues is involved in negative feed-back loops (Brunet *et al*., 1994; Rossomando *et al*., 1994; Xu *et al*., 1999; Sharma *et al*., 2002). This may be a direct consequence of the overall lower level of MEK protein in the iRBC extract; nevertheless, together with the higher phosphorylation of Ser-297 which has a stimulatory effect on MEK1 activity (see below), this is consistent with sustained activation of MEK1/2 in iRBCs. In contrast, the activation loop of MEK3 and MEK4, which are also present in the RBC proteome (Roux-Dalvai *et al*., 2008), are not phosphorylated upon infection (Fig. S6). Furthermore, we observed a slight phosphorylation of MEK4 serine 80 in the infected erythrocytes, a phosphorylation known to lead to inactivation of the kinase (Spillman *et al*., 2007). Thus, direct examination of the phosphorylation status of MEK1 strongly supports the pharmacological data implicating a pathway that selectively involves MEK1 (versus MEK3 and MEK4) in infection of erythrocytes by malaria parasites.

### Activation of a PAK1→MEK1 pathway in infected erythrocytes

**Fig. 5:**
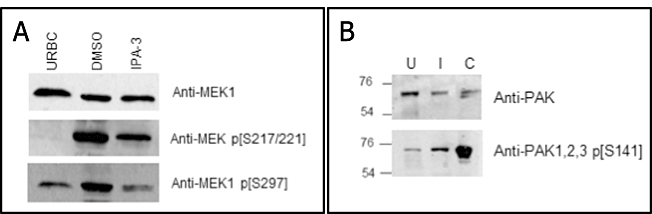
Effects of PAK1 inhibitors on MEK1 phosphorylation and phosphorylation status of host erythrocyte PAK1 in *P. falciparum*-infected cells. A. Effect of the IPA-3 PAK1 inhibitor on MEK1 Ser-297 phosphorylation. Synchronous *P. falciparum* cultures were treated at ring stage with 15 µM IPA-3 (or with the DMSO vehicle only) for 27 h prior to Western blot analysis using the antibodies indicated to the right. URBC, uninfected red blood cells. B. Increased phosphorylation of PAK1 Ser-144 in iRBCs. A Western blot analysis was performed on protein extracts from *in vitro* cultured infected (I) or uninfected (U) RBC ghosts using anti-PAK1 antibody (Cell Signaling; top panel) or anti-phospho-PAK 1/2/3 (Ser-144) (Invitrogen; bottom panel). Lane C (positive control) contains an extract of A673 cells (Santa Cruz).

The only kinase known so far to phosphorylate MEK1 on Ser-297 is the p21-activated protein kinase PAK, several isoforms of which are present in mammalian cells (Slack-Davis *et al*., 2003; Park *et al*., 2007). PAK isoforms have also been shown to be represented in the RBC proteome (Ringrose *et al*., 2008; Roux-Dalvai *et al*., 2008). In fibroblasts, cell interaction with extracellular matrix triggers a pathway leading to phosphorylation of MEK1 Ser-297 by PAK1. This stimulates autophosphorylation of MEK1 on the Ser-217/Ser-221 residues located in the activation loop, leading to activation of the kinase (Park *et al*., 2007). Thus, Ser-297 phosphorylation can be predicted to be accompanied by phosphorylation of Ser-217/Ser-221 in iRBCs. This indeed is what we observed by performing Western blot analysis using a phospho-specific antibody against MEK1 Ser-217/221 (which was not present on the Kinexus KPSS7.0 panel) (Figs 4C and 5A). Furthermore, and consistent with the proposition that phosphorylation of Ser-297 lies upstream of Ser-217/Ser-221 autophosphorylation, treatment with U0126 or PD184352 strongly decreased phosphorylation of Ser-217/221, but not of Ser-297 (Fig. 4C).

The recently described IPA-3 molecule (Fig. 1A) is an allosteric, highly selective inhibitor of PAK1, -2 and -3 (Deacon *et al*., 2008), which was shown to act by binding covalently to the PAK regulatory domain and thereby preventing binding to the upstream activator Cdc42 (Viaud and Peterson, 2009). If activation of host erythrocyte MEK1 is required for parasite survival, and if this activation occurs via PAK-mediated phosphorylation of MEK1 Ser-297, then we would predict PAK inhibition to have similar parasiticidal effects as the MEK inhibitors. We found that the IC_50_ of IPA-3 on parasite growth is close to 2 µM (Fig. S1B). Since the compound has only been identified recently, this value cannot be compared with IC_50_ values in other cellular systems; however, in the original report that first describes the inhibitor, a concentration of 30 µM was used in experiments aimed at measuring cellular effects of IPA-3 (Deacon *et al*., 2008). To verify that IPA-3 treatment did indeed affect MEK1 phosphorylation, we performed a MEK1 p[Ser-297] Western blot analysis of extracts from IPA-3-treated and untreated iRBCs. Treatment with IPA-3 clearly reduces phosphorylation of this MEK1 residue (by two- to threefold as determined by autoradiogram scanning), and as expected, also affects phosphorylation of the serines in the activation loop (Fig. 5A). In other systems, phosphorylation of PAK1 on Ser-144, which lies in a kinase autoinhibitory domain, is known to significantly contribute to its activation (Chong *et al*., 2001). As shown in Fig. 5B, Western blotting using a phospho-specific antibody recognizing this residue gave a stronger signal in iRBCs than in uRBCs, despite a lower amount of PAK1 protein, similar to what we found for MEK1 (see Fig. 4B). This demonstrates that PAK1, like MEK1, is activated in infected erythrocytes, and that MEK1 is activated by PAK1 as a consequence of infection.

Pharmacological (using the IPA-3 inhibitor) and biochemical (using an anti-p[Ser-144] antibody) data thus concur to identify host erythrocyte PAK1 as being, like MEK1, stimulated in infected (versus uninfected) RBCs. Two pieces of evidence establish that both PAK1 and MEK1 function in the same pathway: first, the residue that was found in the Kinexus screen to undergo the highest level of phosphorylation in iRBCs is Ser-297 of MEK1; the only kinase demonstrated so far to use this residue as a substrate is PAK. Second, treatment of parasite cultures with the PAK-specific inhibitor IPA-3 significantly reduced MEK1 Ser-297 phosphorylation.

Taken together, our pharmacological and biochemical data strongly suggest that a PAK1→MEK1 pathway is activated in malaria parasite-infected erythrocytes. This raises exciting questions in two areas: first, how does parasite infection lead to activation of the host PAK1→MEK1 pathway? One possibility is that PAK1 itself may be the target of a parasite kinase. Alternatively, hijacking of the pathway might occur upstream of PAK1. In fibroblasts, PAK1 is activated by interaction with GTP-bound cdc42/Rac1 (Parrini *et al*., 2005), which in turn is stimulated by adhesion of the cell to the extracellular matrix (Park *et al*., 2007). That (i) the PAK inhibitor IPA-3 acts by preventing activation of the kinase through Cdc42 interaction and (ii) IPA-3 treatment reduces MEK1 Ser-297 phosphorylation (Fig. 5A) points to a possible involvement of Rac1/Cdc42 in the pathway. It is attractive to speculate that the extensive remodelling of the erythrocyte plasma membrane following parasite infection may trigger the Rac1/Cdc42→PAK1→MEK1 pathway as described in fibroblasts after interaction of the cell with the extracellular matrix. It is noteworthy that MEK phosphorylation on S297 and on the activation loop is detectable already at the ring stage (Fig. S7), when RBC remodelling is initiated. Second, why is host MEK1 activity required for parasite survival? Erythrocyte MAPK pathways having been implicated in the regulation of transporters (Sartori *et al*., 1999), it may be that MEK1 participates in the activation of the ‘novel permeation pathway’ that permits import of nutrients into the iRBC. Indeed, NPP activation was shown to be susceptible to kinase inhibitors (Baumeister *et al*., 2006). There are a number of other possible effectors of the pathway; identifying substrates of MEK1 in iRBC will be crucial in understanding the essential role this kinase plays in parasite survival. Data in Fig. 4A suggest that infection also affects MEK2; the MEK inhibitors used in our study are effective against both MEK1 and MEK2, and also inhibit MEK5 (Squires *et al*., 2002; Bain *et al*., 2007). It follows that the parasiticidal effect of these molecules may be mediated by several parallel host cell MAPK pathways. We were surprised not to observe a significant phosphorylation of ERK1/2in iRBCs (data not shown), as these MAPKs are the classical substrates of MEK1/2; this may indicate that MEK is diverted to non-conventional substrates in this case.

Even though our data fully support modulation of the host PAK1→MEK1 pathway following infection, we cannot exclude that unidentified parasite-encoded targets contribute to the parasiticidal effect of the inhibitors. However, we checked that U0126 has no detectable effect on the *in vitro* activity of the three *P. falciparum* protein kinases for which weak similarity with mammalian MEKs has been documented: PfPK7 (PlasmoDB identifier PFB0605w), a ‘composite kinase’ whose C-terminal lobe shows maximal homology to MEK3/6 [but whose N-terminal lobe is most similar to fungal PKAs (Dorin *et al*., 2005) and which is not essential for survival of asexual blood-stage parasites (Dorin-Semblat *et al*., 2008)]; Pfnek-1 (PlasmoDB identifier PFL1370w), which, despite an overall high similarity to NIMA kinases, possesses a MEK1-like activation loop (Dorin *et al*., 2001); and PfPK8 (PlasmoDB identifier PFB0150c), for which a distant relation to some STE kinases (the group of kinases that includes MEKs) has been proposed (Anamika *et al*., 2005) (data not shown).

Taken together, the clear evidence provided here that host RBC MEK1 undergoes activating phosphorylation events upon infection, and the high selectivity of the PAK and MEK inhibitors used in this study, strongly point to a crucial role of a host cell PAK-MEK pathway in parasite survival.

### Impact on antimalarial drug discovery

The finding that allosteric MEK and PAK inhibitors have parasiticidal activity has considerable implications in strategies for antimalarial drug discovery. Several protein kinase inhibitors have reached the market, mostly as anticancer agents, in the recent years (Zhang *et al*., 2009), and MAPK pathway components (notably MEKs) are considered as attractive targets for cancer chemotherapy (Sebolt-Leopold and Herrera, 2004). It will be important to verify whether the MEK inhibitors discussed here have a ‘cidal’ or a ‘static’ effect on the parasite; nevertheless, in more general terms, we propose that inhibitors of human kinases (MEK or others) that successfully pass Phase 1 and/or Phase 2 clinical trials, but pass or fail Phase 3 for their original target disease, are evaluated for antimalarial properties. This would considerably reduce the overall discovery/development costs of antimalarials and accelerate the process. A potential problem associated with targeting human enzymes is the toxicity of the compounds; however, this has not prevented the registration of kinase inhibitors for cancer treatment, even though anticancer agents must generally be administered for extended periods of time. In contrast, treatment of severe malaria requires only short treatment periods, limiting the toxicity problem. Importantly, targeting a human enzyme would deprive the parasite of the most straightforward mechanism for emergence of drug resistance, namely the selection of genotypes expressing a mutated, resistant target. That both *P. falciparum* and *P. berghei* are susceptible to MEK inhibitors indicates that reliance on host RBC signalling pathways is widespread across the genus *Plasmodium*, and suggests that other species infecting humans (e.g. *Plasmodium vivax*, which, like *P. berghei*, infects preferentially reticulocytes) are likely to share this feature. Our findings thus define a novel paradigm for strategies towards the discovery and development of antimalarials with a novel mode of action.

## Experimental procedures

### *P. falciparum* culture and hypoxanthine incorporation assay

*Plasmodium falciparum* (clone 3D7) was grown in human erythrocytes as described previously (Dorin *et al*., 1999). U0126 and PD98059 were supplied by Calbiochem. PD184352 was either provided by Prof. Philip Cohen (MRC Protein Phosphorylation Unit, University of Dundee, UK), or synthesized by JAS (see *Supporting information*). IPA-3 was provided by Jeffrey Peterson (Fox Chase Cancer Center, Philadelphia, USA). Inhibitors were dissolved in DMSO, and IC_50_ values were determined by the [^3^H]-hypoxanthine incorporation assay (Desjardins *et al*., 1979). Briefly, asynchronous parasites were aliquoted in 96-well plate at a 0.5% parasitaemia and 5% haematocrit in the presence of the inhibitor (0.04–100 µM). [^3^H]-hypoxanthine (0.1 µCi per well) was added after 24 h and the cells were harvested on a filter mat after a further 24 h of culture. Scintillation liquid was added onto the filter mat and radioactivity counted using a β-scintillation counter. All assays were carried out using untreated parasites with DMSO as controls. Assays were run at least twice in triplicates.

### Soluble protein extracts and Western blots

To obtain infected cell pellets free of uRBCs, asynchronous cultures were passed through a magnetic column (MACS) (130-041-305/MiltenyiBiotec) that retain trophozoite- and schizont-infected cells, but not younger stages or uRBCs. uRBCs and MACS-purified iRBCs were counted on haematimeter, and the same number of cells were sonicated in a lysis buffer containing phosphatase and protease inhibitors (20 mM Tris pH 7.5, 2 mM EGTA, 5 mM EDTA, 30 mM NaF, 40 mM β-glycerophosphate, 20 mM sodium pyrophosphate, 1 mM sodium orthovanadate, 1 mM PMSF, 3 mM benzamidine, 5 µM pepstatin A, 10 µM leupeptin and 0.5% Triton X-100). Lysates were cleared by centrifugation at 10 000 *g* for 15 min at 4°C.

For Western blot analysis, iRBC and uRBC samples were normalized by cell number. Polyacrylamide gel electrophoresis (SDS-PAGE) and transfer were performed using standard procedure. The nitrocellulose membrane was blocked for 1 h in Tris-buffered saline (pH 7.6) (TBS) containing 0.1% Tween-20 with 5% w/v non-fat dry milk and exposed overnight at 4°C to the primary antibody [1:1000 dilution in blocking buffer for anti-MEK1 (Biosource, Invitrogen) and the following anti-MEK1 phospho-specific antibodies: anti-p[S217–S221] from Calbiochem, anti-p[S217–S221] from Santa Cruz and anti-p[S297] from BioSource]. After washing, the membrane was incubated for 1 h at room temperature with 1:1000 anti-rabbit horseradish peroxidase-conjugated secondary antibody (Sigma). Detection was performed using the ECL Chemiluminescence system from Perkin-Elmer following the manufacturer's recommendations.

For experiments performed at Kinexus, extracts were prepared according to Kinexus recommendations and shipped on dry ice.

### Protein extraction and mass spectrometry analysis

Uninfected erythrocytes were lysed with 150 mM NaCl, 5 mM EDTA, 50 mM Tris pH 8.0, 1% Triton X-100 and centrifuged at 13 000 r.p.m. for 20 min at 4°C. The supernatant was used for immunoprecipitation using either mouse anti-MEK1 agarose-conjugated (Santa-Cruz Biotechnology) or mouse IgG agarose-conjugated (Santa Cruz) as a control for 4 h on a wheel at 4°C. Beads were washed four times with PBS mixed with 4× Laemelli and boil before electrophoresis of duplicate gels. One gel was Coomassie stained while the other was blotted onto a nitrocellulose membrane. The presence of MEK1 was detected as described previously. Spots corresponding to immunoreactive regions of the blot were excised from the Coomassie-stained gel (Fig. S4A). After in-gel digestion, tryptic peptides were separated by nanoflowrpHPLC and analysed on an LTQ-Orbitrap XL mass spectrometer (Thermo Fisher Scientific). Data search was performed using Mascot 2.2 (Matrix Science) in Proteome Discoverer v.1.1 against a concatenated database consisting of the Swiss-Prot v.57.13 database and the reversed-sequence version of the same database. Data were visualized using Scaffold 3 software.

### Preparation of ghosts and protein extraction

Proteins of ghosts from uninfected and infected erythrocytes were extracted according to Blisnick *et al*. (2000). Uninfected erythrocytes and infected erythrocytes were incubated 30 min at 4°C in 1:10 and 1:5, respectively, diluted RPMI and spun 45 min at 5100 r.p.m. at 4°C. The upper layer containing the haemoglobin was discarded. The bottom layer was washed three times 3 min in lysis buffer at 13 000 r.p.m. at 4°C. Proteins were extracted using 50 mM Tris pH 8.0, 300 mM NaCl, 0.1 mM EDTA, 1% Triton X-100 supplemented with proteases and phosphatases inhibitors and subjected to Western blot analysis as described above.

### Determination of IC_50_ values on *P. berghei* blood-stage proliferation

*In vitro* drug susceptibility test was performed in standard short-term cultures of synchronized *P. berghei* blood stages. See *Supporting information* for details.

## Supporting information

**Fig. S1.** Effect of allosteric inhibitors of host erythrocyte kinases on *P. falciparum* proliferation. The [3H]-hypoxanthine incorporation was used (see *Experimental procedures* for details). Assays were run twice in triplicates and error bars correspond to SEM.

A. MEK inhibitors. The range of the concentrations used was 0.412–100 µM for U0126 and PD98059, and 0.041–20 µM for PD184352.

B. PAK inhibitor IPA-3.

**Fig. S2.** MEK inhibitors do not affect egress or invasion. Schizont-enriched cultures (0.5% parasitaemia) were treated with MEK inhibitors (U0126 and PD184352, 20 µM) or the vehicle (DMSO) as negative control. Aliquots were smeared at the time of treatment (labelled ‘T0’, left) and 12 h post treatment (’T12’, right), and the parasitaemia examined. Cell numbers were obtained from microscopic examination of 10 fields for each time point. For each condition, assays were performed in triplicates. The treatment did not decrease the number and proportion of rings following re-invasion.

**Fig. S3.** Determination of the IC_50_ values of MEK inhibitors on *P. berghei* blood-stage proliferation. *In vitro* drug susceptibility test was performed in standard short-term cultures of synchronized *P. berghei* blood stages. Cultured and purified schizonts/merozoites, obtained by Nycodenz density gradient purification were injected i.v. into the tail vein of a rat. Injected merozoites invade within 4 h after injection and newly infected blood was collected from the rat by heart puncture at 4 h after the injection of the purified schizonts/merozoites. Infected blood was washed once (450 *g*, 8 min) with complete culture medium (RPMI + 25% FCS) followed by mixing of infected erythrocytes with serial solutions of the drugs in complete culture medium and incubated in 24-well plates in triplicate at a final concentration of 1% at 35°C for 24 h thus allowing the ring forms/young trophozoites to develop into mature schizonts. Parasite development was analysed by FACS analysis after staining of the parasites with the DNA-specific dye Hoechst33258. The cell suspension (0.5 ml) of each well was transferred to an Eppendorf tube and cells were pelleted by centrifugation (13 000 *g* for 5 s). After removal of the culture medium, the cells were fixed in 500 µl of a 0.25% glutaraldehyde/PBS solution and stored at 4°C until staining. Prior to flow-cytometric analysis, the cells were stained in 500 µl of a 2 µM Hoechst33258 solution in PBS for 1 h at 37°C. Stained cells were analysed using a MACSQuant analyser (Miltenyi Biotec, Germany). UV excitation of Hoechst33258 dye was performed with a violet laser (450/50 nm) and the (infected) erythrocyte population was selected by gating on forward/sidelight scatter. The fluorescence intensity of a total of 50 000 cells per sample was measured for each sample. The mean fluorescence intensity of the infected erythrocyte population, which is proportional to the mean DNA content of the parasites, was calculated for each drug concentration in triplicate. For calculation of the growth inhibitory curves, the mean fluorescence intensity value of samples with the highest drug concentration (i.e. with maximum inhibition of growth) was subtracted from the mean fluorescence intensity value of the samples with the other drug concentrations and the control samples without drug. The mean fluorescence intensity value of the control samples was set at 100% and the mean fluorescence value of the highest drug concentration was set at 0% for calculation of the percentage of inhibition. Growth inhibitory curves were constructed in MS Excel.

**Fig. S4.** Immunoprecipitation and mass spectrometry analysis of erythrocytic MEK1.

A. Coomassie-stained gel of total-cell extracts from uRBCs after immunoprecipitation using either mouse anti-MEK1 agarose-conjugated (lane 1) or mouse IgG agarose-conjugated as a control (lane 2).

B. List of the 24 unique peptides leading to the identification of MEK1 following mass spectrometry analysis.

**Fig. S5.** Western blot of total-cell extracts from uRBCs (lane 1) and iRBCs (lane 2) probed with an anti-phospho-MEK1 p[S297] antibody. This experiment was performed at Kinexus.

**Fig. S6.** Phosphorylation status of host cell MEK1, MEK3 and MEK4 in *P. falciparum*-infected erythrocytes. Quantification of the signal of MEK1 S297, MEK3 S189, MEK4 S257 + T261 and MEK4 S80 in uninfected erythrocytes (white columns) and infected erythrocytes (black columns) from a second Kinexus experiment.

**Fig. S7.** Western blot of protein extracts from *in vitro* cultured infected RBCs ghosts at rings, trophozoites and schizonts stages (as determined by Giemsa staining) probed with the antibodies indicated to the right.

## References

[b1] Anamika, Srinivasan N, Krupa A (2005). A genomic perspective of protein kinases in *Plasmodium falciparum*. Proteins.

[b2] Bain J, Plater L, Elliott M, Shpiro N, Hastie CJ, McLauchlan H (2007). The selectivity of protein kinase inhibitors: a further update. Biochem J.

[b3] Baumeister S, Winterberg M, Duranton C, Huber SM, Lang F, Kirk K, Lingelbach K (2006). Evidence for the involvement of *Plasmodium falciparum* proteins in the formation of new permeability pathways in the erythrocyte membrane. Mol Microbiol.

[b4] Blisnick T, Morales Betoulle ME, Barale JC, Uzureau P, Berry L, Desroses S (2000). Pfsbp1, a Maurer's cleft *Plasmodium falciparum* protein, is associated with the erythrocyte skeleton. Mol Biochem Parasitol.

[b5] Brunet A, Pages G, Pouyssegur J (1994). Growth factor-stimulated MAP kinase induces rapid retrophosphorylation and inhibition of MAP kinase kinase (MEK1). FEBS Lett.

[b6] Chong C, Tan L, Lim L, Manser E (2001). The mechanism of PAK activation. Autophosphorylation events in both regulatory and kinase domains control activity. J Biol Chem.

[b7] Davis RJ (2000). Signal transduction by the JNK group of MAP kinases. Cell.

[b8] Deacon SW, Beeser A, Fukui JA, Rennefahrt UE, Myers C, Chernoff J, Peterson JR (2008). An isoform-selective, small-molecule inhibitor targets the autoregulatory mechanism of p21-activated kinase. Chem Biol.

[b9] DeSilva DR, Jones EA, Favata MF, Jaffee BD, Magolda RL, Trzaskos JM, Scherle PA (1998). Inhibition of mitogen-activated protein kinase kinase blocks T cell proliferation but does not induce or prevent anergy. J Immunol.

[b10] Desjardins RE, Canfield CJ, Haynes JD, Chulay JD (1979). Quantitative assessment of antimalarial activity *in vitro* by a semiautomated microdilution technique. Antimicrob Agents Chemother.

[b11] Dorin D, Alano P, Boccaccio I, Ciceron L, Doerig C, Sulpice R (1999). An atypical mitogen-activated protein kinase (MAPK) homologue expressed in gametocytes of the human malaria parasite *Plasmodium falciparum*. Identification of a MAPK signature. J Biol Chem.

[b12] Dorin D, Le Roch K, Sallicandro P, Alano P, Parzy D, Poullet P (2001). Pfnek-1, a NIMA-related kinase from the human malaria parasite *Plasmodium falciparum* biochemical properties and possible involvement in MAPK regulation. Eur J Biochem.

[b13] Dorin D, Semblat JP, Poullet P, Alano P, Goldring JP, Whittle C (2005). PfPK7, an atypical MEK-related protein kinase, reflects the absence of classical three-component MAPK pathways in the human malaria parasite *Plasmodium falciparum*. Mol Microbiol.

[b14] Dorin-Semblat D, Quashie N, Halbert J, Sicard A, Doerig C, Peat E (2007). Functional characterization of both MAP kinases of the human malaria parasite *Plasmodium falciparum* by reverse genetics. Mol Microbiol.

[b15] Dorin-Semblat D, Sicard A, Doerig C, Ranford-Cartwright L, Doerig C (2008). Disruption of the PfPK7 gene impairs schizogony and sporogony in the human malaria parasite *Plasmodium falciparum*. Eukaryot Cell.

[b16] Graewe S, Retzlaff S, Struck N, Janse CJ, Heussler VT (2009). Going live: a comparative analysis of the suitability of the RFP derivatives RedStar, mCherry and tdTomato for intravital and *in vitro* live imaging of *Plasmodium* parasites. Biotechnol J.

[b17] Haldar K, Mohandas N (2007). Erythrocyte remodeling by malaria parasites. Curr Opin Hematol.

[b18] Harrison T, Samuel BU, Akompong T, Hamm H, Mohandas N, Lomasney JW, Haldar K (2003). Erythrocyte G protein-coupled receptor signaling in malarial infection. Science.

[b19] Heussler V, Sturm A, Langsley G (2006). Regulation of host cell survival by intracellular *Plasmodium* and *Theileria* parasites. Parasitology.

[b20] Manno S, Takakuwa Y, Mohandas N (2005). Modulation of erythrocyte membrane mechanical function by protein 4.1 phosphorylation. J Biol Chem.

[b21] Ohren JF, Chen H, Pavlovsky A, Whitehead C, Zhang E, Kuffa P (2004). Structures of human MAP kinase kinase 1 (MEK1) and MEK2 describe novel noncompetitive kinase inhibition. Nat Struct Mol Biol.

[b22] Park ER, Eblen ST, Catling AD (2007). MEK1 activation by PAK: a novel mechanism. Cell Signal.

[b23] Parrini MC, Matsuda M, de Gunzburg J (2005). Spatiotemporal regulation of the Pak1 kinase. Biochem Soc Trans.

[b24] Prudencio M, Rodrigues CD, Hannus M, Martin C, Real E, Goncalves LA (2008). Kinome-wide RNAi screen implicates at least 5 host hepatocyte kinases in *Plasmodium* sporozoite infection. PLoS Pathog.

[b25] Raman M, Chen W, Cobb MH (2007). Differential regulation and properties of MAPKs. Oncogene.

[b26] Ringrose JH, van Solinge WW, Mohammed S, O'Flaherty MC, van Wijk R, Heck AJ, Slijper M (2008). Highly efficient depletion strategy for the two most abundant erythrocyte soluble proteins improves proteome coverage dramatically. J Proteome Res.

[b27] Rossomando AJ, Dent P, Sturgill TW, Marshak DR (1994). Mitogen-activated protein kinase kinase 1 (MKK1) is negatively regulated by threonine phosphorylation. Mol Cell Biol.

[b28] Roux-Dalvai F, Gonzalez de Peredo A, Simo C, Guerrier L, Bouyssie D, Zanella A (2008). Extensive analysis of the cytoplasmic proteome of human erythrocytes using the peptide ligand library technology and advanced mass spectrometry. Mol Cell Proteomics.

[b29] Sartori M, Ceolotto G, Semplicini A (1999). MAPKinase and regulation of the sodium-proton exchanger in human red blood cell. Biochim Biophys Acta.

[b30] Sebolt-Leopold JS, Herrera R (2004). Targeting the mitogen-activated protein kinase cascade to treat cancer. Nat Rev Cancer.

[b31] Sharma P, Veeranna, Sharma M, Amin ND, Sihag RK, Grant P (2002). Phosphorylation of MEK1 by cdk5/p35 down-regulates the mitogen-activated protein kinase pathway. J Biol Chem.

[b32] Singh AP, Buscaglia CA, Wang Q, Levay A, Nussenzweig DR, Walker JR (2007). *Plasmodium* circumsporozoite protein promotes the development of the liver stages of the parasite. Cell.

[b33] Slack-Davis JK, Eblen ST, Zecevic M, Boerner SA, Tarcsafalvi A, Diaz HB (2003). PAK1 phosphorylation of MEK1 regulates fibronectin-stimulated MAPK activation. J Cell Biol.

[b34] Spillman MA, Lacy J, Murphy SK, Whitaker RS, Grace L, Teaberry V (2007). Regulation of the metastasis suppressor gene MKK4 in ovarian cancer. Gynecol Oncol.

[b35] Squires MS, Nixon PM, Cook SJ (2002). Cell-cycle arrest by PD184352 requires inhibition of extracellular signal-regulated kinases (ERK) 1/2 but not ERK5/BMK1. Biochem J.

[b36] Viaud J, Peterson JR (2009). An allosteric kinase inhibitor binds the p21-activated kinase autoregulatory domain covalently. Mol Cancer Ther.

[b37] Ward P, Equinet L, Packer J, Doerig C (2004). Protein kinases of the human malaria parasite *Plasmodium falciparum*: the kinome of a divergent eukaryote. BMC Genomics.

[b38] Xu B, Wilsbacher JL, Collisson T, Cobb MH (1999). The N-terminal ERK-binding site of MEK1 is required for efficient feedback phosphorylation by ERK2 *in vitro* and ERK activation *in vivo*. J Biol Chem.

[b39] Zhang J, Yang PL, Gray NS (2009). Targeting cancer with small molecule kinase inhibitors. Nat Rev Cancer.

