# Supplementary material for: Activation of a PAK-MEK signalling pathway in malaria parasite-infected erythrocytes
Source: Cell Microbiol. 2011 Jun;13(6):836–45. doi: 10.1111/j.1462-5822.2011.01582.x (PMC3123749; doi:10.1111/j.1462-5822.2011.01582.x)
Supplement: Supplementary file 1 [file cmi0013-0836-SD1.pdf]

LEGEND TO SUPPORTING FIGURES

Figure S1. Effect of allosteric inhibitors of host erythrocyte kinases on *P. falciparum* proliferation.

The [^{3}H]-hypoxanthine incorporation was used (see Materials and Methods for details). Assays were run twice in triplicates and error bars correspond to SEM.

A. MEK inhibitors. The range of the concentrations used was 0.412 to 100μM for U0126 and PD98059, and 0.041 to 20μM for PD184352.
B. PAK inhibitor IPA-3.

Figure S2. MEK inhibitors do not affect egress or invasion.

Schizont-enriched cultures (0.5% parasitemia) were treated with MEK inhibitors (U0126 and PD184352, 20 μM) or the vehicle (DMSO) as negative control. Aliquots were smeared at the time of treatment (labelled "T0", left) and 12 hours post treatment ("T12", right), and the parasitemia examined. Cell numbers were obtained from microscopic examination of 10 fields for each time point. For each condition, assays were performed in triplicates. The treatment did not decrease the number and proportion of rings following re-invasion.

Figure S3. Determination of the IC_{50} values of MEK inhibitors on *P. berghei* blood stage proliferation.

In vitro drug susceptibility test was performed in standard short-term cultures of synchronised *P. berghei* blood stages. Cultured and purified schizonts/merozoites, obtained by Nycodenz density gradient purification were injected i.v. into the tail vein of a rat. Injected merozoites invade within 4 h after injection and newly infected blood was collected from the rat by heart puncture at 4 h after the injection of the purified schizonts/merozoites. Infected blood was washed once (450 g, 8 min) with complete culture medium (RPMI + 25% FCS)

followed by mixing of infected erythrocytes with serial solutions of the drugs in complete culture medium and incubated in 24 well plates in triplicate at a final concentration of 1% at 35°C for 24 hours thus allowing the ring forms/young trophozoites to develop into mature schizonts. Parasite development was analysed by FACS analysis after staining of the parasites with the DNA-specific dye Hoechst33258. The cell suspension (0.5 ml) of each well was transferred to an Eppendorf tube and cells were pelleted by centrifugation (13,000g for 5 s). After removal of the culture medium, the cells were fixed in 500 µl of a 0.25% glutaraldehyde/PBS solution and stored at 4°C until staining. Prior to flow-cytometric analysis, the cells were stained in 500 µl of a 2 µM Hoechst33258 solution in PBS for 1 h at 37°C. Stained cells were analysed using a MACSQuant analyser (Miltenyi Biotec, Germany). UV excitation of Hoechst33258 dye was performed with a violet laser (450/50 nm) and the (infected) erythrocyte population was selected by gating on forward/sidelight scatter. The fluorescence intensity of a total of 50,000 cells per sample was measured for each sample. The mean fluorescence intensity of the infected erythrocyte population, which is proportional to the mean DNA content of the parasites, was calculated for each drug concentration in triplicate. For calculation of the growth inhibitory curves, the mean fluorescence intensity value of samples with the highest drug concentration (i.e. with maximum inhibition of growth) was subtracted from the mean fluorescence intensity value of the samples with the other drug concentrations and the control samples without drug. The mean fluorescence intensity value of the control samples was set at 100% and the mean fluorescence value of the highest drug concentration was set at 0% for calculation of the percentage of inhibition. Growth inhibitory curves were constructed in MS Excel.

Figure S4. Immunoprecipitation and mass spectrometry analysis of erythrocytic MEK1.

A. Coomassie stained gel of total cell extracts from uRBCs after immunoprecipitation using either mouse anti-MEK1 agarose-conjugated (lane1) or mouse IgG agarose-conjugated as a control (lane 2).

B. List of the 24 unique peptides leading to the identification of MEK1 following mass spectrometry analysis.

Figure S5.

Western blot of total cell extracts from uRBCs (lane1) and iRBCs (lane 2) probed with an anti phospho-MEK1 p[S297] antibody. This experiment was performed at Kinexus.

Figure S6. Phosphorylation status of host cell MEK1, MEK3 and MEK4 in *P. falciparum*-infected erythrocytes. Quantification of the signal of MEK1 S297, MEK3 S189, MEK4 S257 + T261 and MEK4 S80 in uninfected erythrocytes (white columns) and infected erythrocytes (black columns) from a second Kinexus experiment.

Figure S7.

Western blot of protein extracts from in vitro cultured infected RBCs ghosts at rings, trophozoites and schizonts stages (as determined by Giemsa staining) probed with the antibodies indicated to the right.

Figure S1

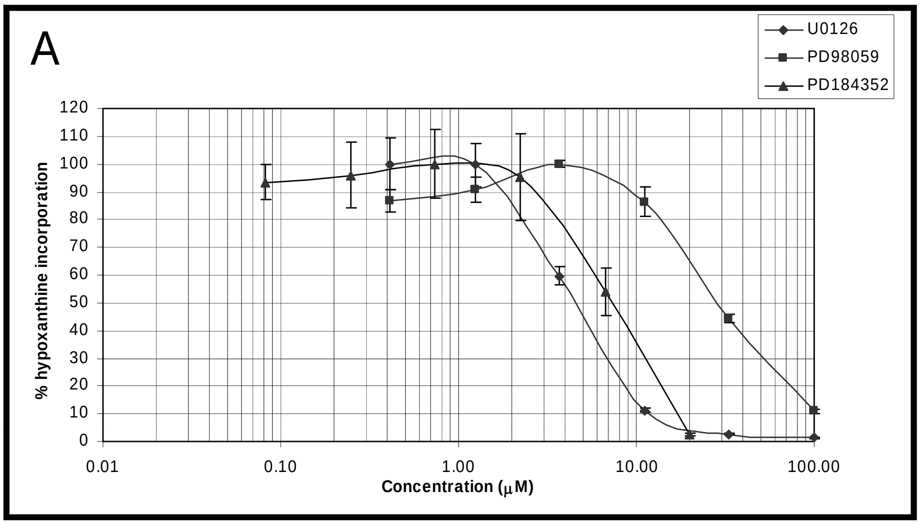
A
U0126
PD98059
PD184352
% hypoxanthine incorporation
120
110
100
90
80
70
60
50
40
30
20
10
0
0.01
0.10
1.00
10.00
100.00
Concentration (μM)

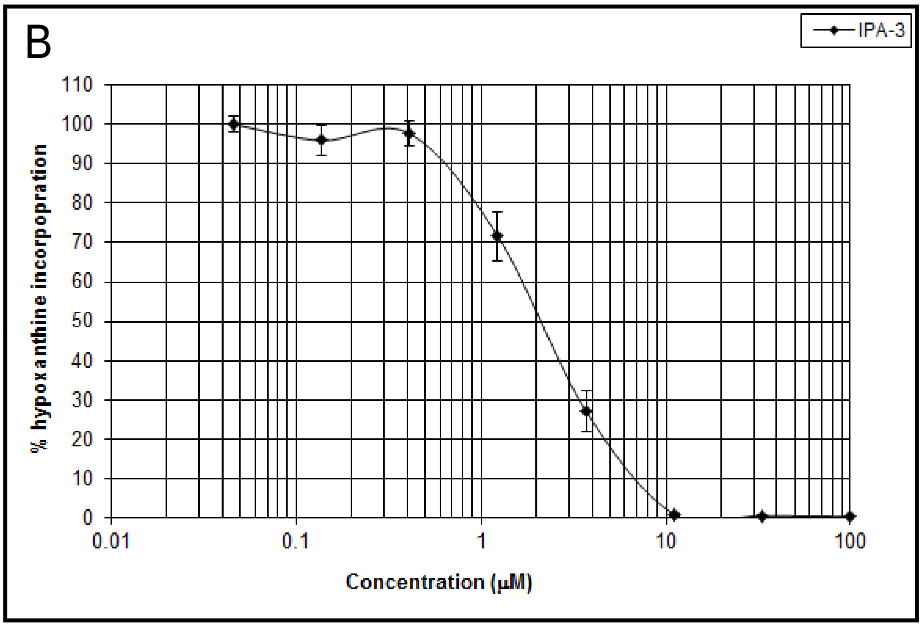
B
IPA-3
% hypoxanthine incorpopration
110
100
90
80
70
60
50
40
30
20
10
0
0.01
0.1
1
10
100
Concentration (μM)

Figure S2

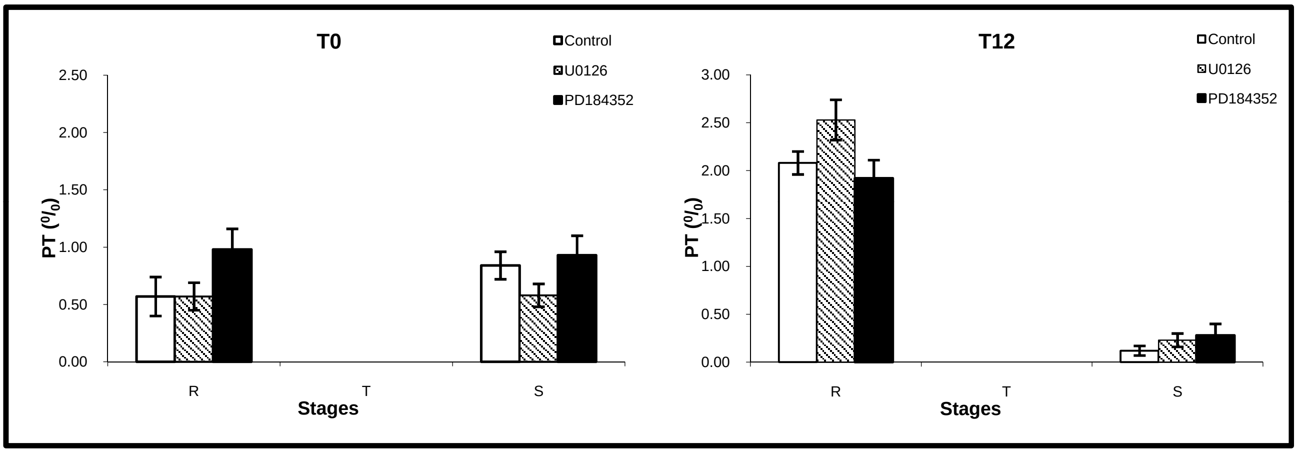

Figure S3

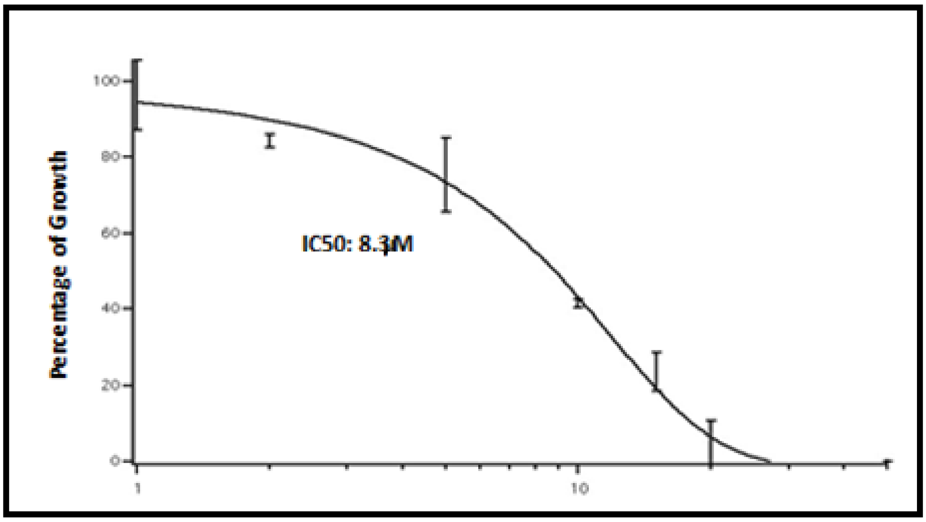

Figure S4

A

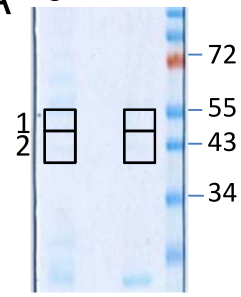

B

Sequence	Mascot Ion Score	Modifications	Observed m/z	Actual peptide Mass	Charge	Delta ppm	Start	Stop
(K)KLEELELDEQQR(K)	92.64		765.3956	1'528.78	2	1.243	36	47
(K)LEELELDEQQR(K)	65.96		701.3472	1'400.68	2	0.1683	37	47
(K)LEELELDEQQRK(R)	29.61		510.5989	1'528.77	3	-5.929	37	48
(R)KRLEAFLTQK(Q)	32.4		411.917	1'232.73	3	2.517	48	57
(K)RLEAFLTQK(Q)	40.18		553.3234	1'104.63	2	1.612	49	57
(R)LEAFLTQK(Q)	35.26		475.2725	948.5293	2	1.209	50	57
(K)VGELKDDDFEK(I)	59.42		647.8132	1'293.61	2	1.162	60	70
(K)ISELGAGNGGVVFK(V)	81.3		674.3685	1'346.72	2	1.264	71	84
(K)VSHKPSGLVmAR(K)	32.36	Oxidation (+16)	433.2412	1'296.70	3	2.259	85	96
(R)KLIHLEIKPAIR(N)	51.14		477.6458	1'429.91	3	0.327	97	108
(K)LIHLEIKPAIR(N)	43.66		434.9479	1'301.82	3	1.28	98	108
(R)IPEQILGK(V)	39.94		449.275	896.5344	2	1.124	161	168
(K)VSIAVIK(G)	34.13		365.2479	728.4801	2	0.5409	169	175
(K)GLTYLR(E)	18.03		361.7139	721.4122	2	-12.22	176	181
(R)DVKPSNILVNSR(G)	85.61		671.3795	1'340.74	2	1.565	190	201
(K)LcDFGVSGQLIDSmANSFVGTR(S)	78.11	Carbamidomethyl (+57), Oxidation (+16)	1'195.56	2'389.11	2	2.081	206	227
(K)LcDFGVSGQLIDSMANSFVGTR(S)	72.25	Carbamidomethyl (+57)	792.0478	2'373.12	3	4.4	206	227
(R)LQGTHYSVQSDIWSmGLSLVEmAVGR(Y)	18.02	Oxidation (+16), Oxidation (+16)	966.1409	2'895.40	3	3.29	235	260
(R)YPIPPPDAK(E)	46.87		499.2721	996.53	2	0.28	260	269
(K)ELELmFGcQVEGDAAETPPRPR(T)	69.81	Oxidation (+16), Carbamidomethyl (+57)	840.0632	2'517.17	3	1.135	270	291
(K)LPSGVFSLEFQDFVNK(C)	74.56		913.972	1'825.93	2	1.779	325	340
(K)QLmVHAFIK(R)	25.74	Oxidation (+16)	551.8082	1'101.60	2	0.09262	354	362
(K)RSDAEEVDFAGWLcSTIGLNQPSTPTHAAGV(-)	67.7	Carbamidomethyl (+57)	1'096.19	3'285.54	3	1.86	363	393
(R)SDAEEVDFAGWLcSTIGLNQPSTPTHAAGV(-)	60.19	Carbamidomethyl (+57)	1'044.15	3'129.44	3	1.848	364	393

Figure S5

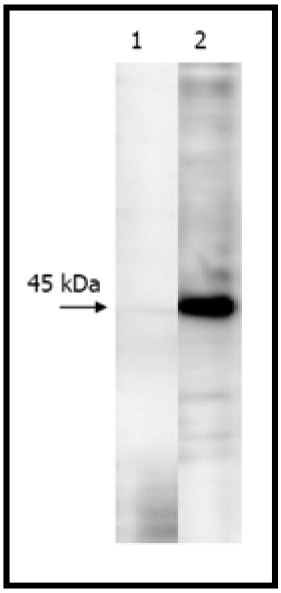

Figure S6

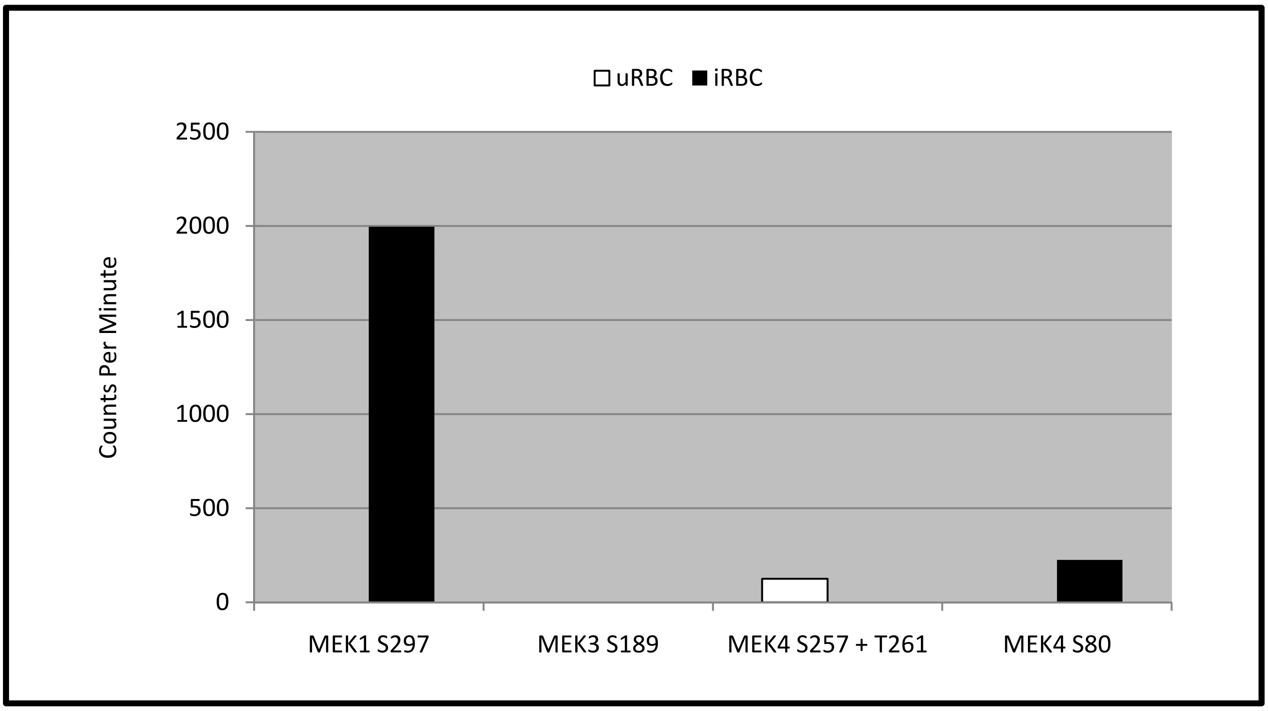

Figure S7

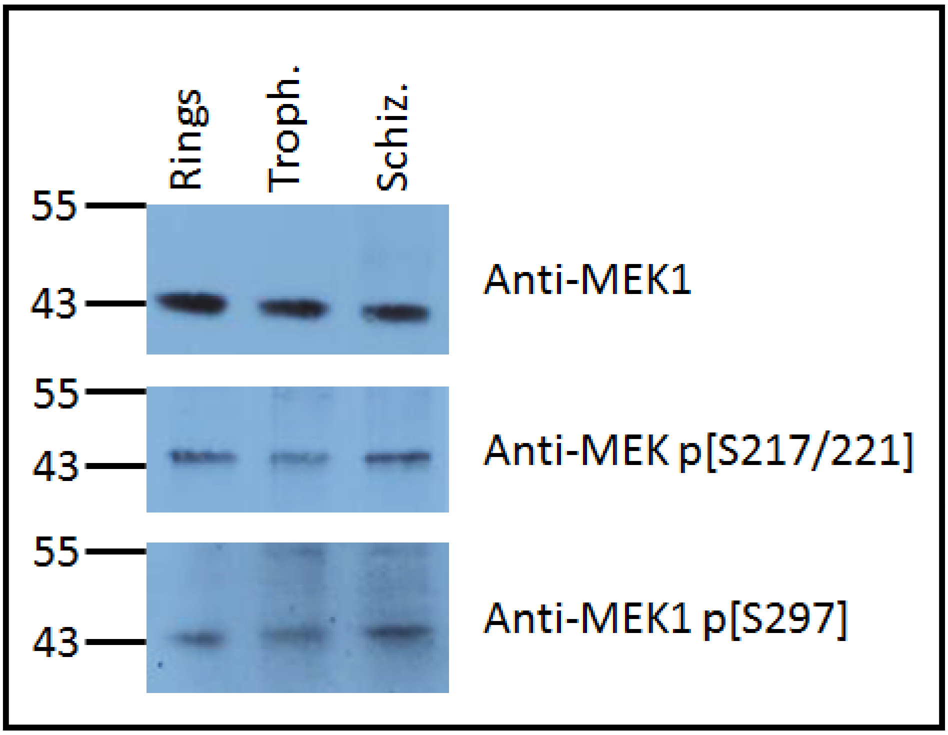
Rings
Troph.
Schiz.
55
43
Anti-MEK1
55
43
Anti-MEK p[S217/221]
55
43
Anti-MEK1 p[S297]

ADDITIONAL SUPPORTING INFORMATION

Synthesis of PD184352

2-(2-Chloro-4-iodophenylamino)-3,4-difluorobenzoic acid (870 mg, 2.13 mmol) was dissolved in dry THF (10 mL), to which was added pyridine (0.67 mL, 8.52 mmol), followed by pentafluorophenyl trifluoroacetate (0.39 mL, 2.34 mmol). This mixture was stirred for 1 h. at room temperature, then O-(cyclopropylmethyl)hydroxylamine hydrochloride (526 mg, 4.26 mmol) was added and stirring continued for a further 2 h. The reaction mixture was then partitioned between 1 M HCl (100 mL) and EtOAc (200 mL). The organic layer was washed with 1 M HCl (2x100 mL), water (100 mL) and brine (100 mL), then dried (Na2SO4). The solvent was removed under reduced pressure and the resulting residue purified by flash column chromatography on silica gel (10% EtOAc in hexanes as eluent) to give 2-(2-chloro-4-iodophenylamino)-N-(cyclopropylmethoxy)-3,4-difluorobenzamide (PD184352) as a white solid (776 mg, 76%), m.p.179-180oC (lit. 172.5-173.5oC). 1H NMR in agreement with the literature (4). Anal. calcd. for C17H14ClF2N2O2 C; 42.7, H; 3.0, N; 5.8. Found C; 42.7; H; 3.0, N; 5.8. HPLC purity 98.8%.
